# Effect of YAP on an Immortalized Periodontal Ligament Stem Cell Line

**DOI:** 10.1155/2019/6804036

**Published:** 2019-04-01

**Authors:** Xiyan Chen, Qi Wang, Ke Gu, Aonan Li, Xucheng Fu, Ying Wang, Weiting Gu, Yong Wen

**Affiliations:** ^1^Shandong Provincial Key Laboratory of Oral Tissue Regeneration, School of Stomatology, Shandong University, Jinan, Shandong Province, China; ^2^School of Stomatology, Shandong University, Jinan, Shandong Province, China; ^3^Ningbo Stomatology Hospital, Zhejiang, China; ^4^Qilu Hospital of Shandong University, Jinan, Shandong Province, China

## Abstract

**Objective:**

To establish an immortalized human periodontal ligament stem cell line (hPDLSC) and investigate whether and how YAP mediates the establishment of the stem cell line.

**Methods:**

Primary hPDLSCs were cultured and transfected with lentivirus containing the telomerase reverse transcriptase (TERT) gene. The expression of TERT was detected via the polymerase chain reaction (PCR) and real-time quantitative PCR (RT-PCR). Flow cytometry was employed to detect surface markers of hPDLSCs and TERT-hPDLSCs. The cell counting kit-8 (CCK-8) and 5-ethynyl-2'-deoxyuridine (EdU) methods were used to examine the proliferation ability of the cells. Flow cytometry and TUNEL staining were employed to examine the cell apoptosis rate. The *β*-galactosidase staining assay was used to assess the rate of cell senescence. The osteogenic differentiation ability of the cells was detected via alkaline phosphatase (ALP) staining and Alizarin red staining assays. BALB/c mice were employed to determine the tumorigenicity of TERT-hPDLSCs. The expression levels of YAP and other proteins in the Hippo signaling pathway were detected by Western blotting. Verteporfin was used to inhibit the binding of YAP to the downstream target gene TEAD.

**Results:**

TERT-hPDLSCs showed stable high expression of TERT, even at the thirtieth passage after transfection with lentivirus containing the TERT gene. Compared with primary hPDLSCs, TERT-hPDLSCs exhibited a stronger proliferation ability and lower cell apoptosis and senescence rates while maintaining the same osteogenetic differentiation ability as primary hPDLSCs. The transfection of hPDLSCs with lentivirus containing the TERT gene did not lead to tumorigenesis in nude mice. The Hippo signaling pathway was inactivated in TERT-hPDLSCs compared to hPDLSCs. When treated with verteporfin, the proliferation of TERT-hPDLSCs decreased, while the apoptosis and senescence rates of these cells increased. However, TERT-hPDLSCs still showed a stronger proliferation ability and lower cell apoptosis and senescence rates than hPDLSCs treated with verteporfin at the same concentration.

**Conclusions:**

Overexpression of TERT in hPDLSCs resulted in the successful establishment of an immortalized periodontal ligament stem cell line. TERT may regulate the biological characteristics of hPDLSCs through the Hippo/YAP signaling pathway. hPDLSCs could be a feasible resource for stem cell research and a promising resource for stem cell therapy.

## 1. Introduction

Periodontal disease is a common type of oral disease in humans. Periodontal disease may cause damage or defects of the teeth and periodontal support tissues, such as the periodontal ligaments, mouth mucosa, and alveolar bone. The teeth will then begin to shift, eventually falling out. This type of disease can impair human health. When teeth become loose or even fallout, some treatment must generally be applied for the missing teeth. Furthermore, periodontal support tissues, such as the alveolar bone, may be damaged. Thus, the dentist must first restore the missing alveolar bone. Periodontal support tissue reconstruction mainly depends on machinery, drugs, or guided tissue regeneration techniques [1]. Seed cells are the basis of periodontal tissue regeneration. Human periodontal ligament stem cells (hPDLSCs) can differentiate not only into osteoblast-like cells but also into fibroblast-like cells. hPDLSCs can differentiate into both bone-like tissue and tissue similar to the natural periodontal membrane connective tissue. PDLSCs are ideal seed cells in periodontal tissue regeneration [2–4]. Thus, an increasing number of studies on PDLSCs have been carried out in recent years, requiring the production of more hPDLSCs. However, the use of hPDLSCs is usually limited because many types of primary stem cells, including hPDLSCs, can become senescent and possess a relatively short replicative life span when cultured *in vitro*. Thus, it is difficult to obtain a sufficient number of hPDLSCs. Many studies have been carried out in an attempt to delay cellular senescence *in vitro* to establish an immortalized hPDLSC line [5, 6].

Additionally, there has been keen interest in research aimed at controlling telomerase reverse transcriptase (TERT), which is a composite template of telomerase [7]. Telomerase is an RNA-dependent DNA polymerase that is responsible for maintaining telomere length, protecting chromosomes from degradation, end-end fusion, rearrangements, and chromosome loss to resist senescence [8–11]. Cell senescence is a type of cell cycle arrest. Cell cycle arrest limits the proliferative potential of cells when they are cultured *in vitro*, which is referred to as DNA replication senescence. The program of cellular senescence is related to DNA damage, telomere dysfunction, oxidative stress, and some other stimuli [12, 13]. The length of the telomere will gradually be shortened as cells divide. When telomere length is reduced to a certain degree, cells will enter a senescent stage, in which cell proliferation stops, and the rate of apoptotic cells increases. Stem cells gradually lose the ability to undergo osteoblastic differentiation and other multipotential differentiation abilities. Overexpression of TERT has been proven to be a promising strategy for overcoming replicative senescence. Some reports have shown that during this process, there are many other signaling pathways involved in the regulation of cellular senescence [14, 15].

The Hippo pathway is comprised of kinase-adaptor protein complexes. The core machinery of the Hippo pathway consists of a kinase relay module and a transcriptional module [16]. When the pathway is activated, Hippo kinase phosphorylates and activates Wts kinase, which in turn phosphorylates and inactivates the transcriptional coactivator YAP. YAP interacts with TEA domain (TEAD) family transcription factors to induce cell cycle-promoting and antiapoptotic gene transcription. Disorders of the Hippo pathway lead to organ overgrowth. In other words, when the kinase module is “on,” it keeps YAP from entering the nucleus, and YAP is then partially degraded; when it is “off” (the transcriptional module becomes active), YAP enters the nucleus and plays a regulatory role in combination with downstream target genes such as TEAD. Previous studies have shown that the Hippo/YAP signaling pathway plays an important role not only in the regulation of cell proliferation and apoptosis but also in cellular senescence [17]. The Hippo/YAP pathway is unique in that it does not involve an extracellular ligand or a dedicated plasma membrane receptor. Therefore, it must be activated by other pathways or proteins. There have also been many studies confirming that the Hippo/YAP pathway can engage in crosstalk with other pathways, such as the Wnt/*β*-catenin signal pathway [18, 19]. Both TERT and Hippo/YAP can regulate the proliferation and senescence of human stem cells. However, it remains to be determined whether there are relationships between the two mechanisms and how crosstalk occurs between Hippo/YAP and TERT to coregulate cell senescence [20].

This study was designed to establish an immortalized hPDLSC model *in vitro* through transfecting hPDLSCs with lentivirus containing the TERT gene. The purpose was to investigate the regulatory function of the Hippo/YAP signaling pathway in this immortalized hPDLSC line.

## 2. Materials and Methods

### 2.1. Cell Culture

All human tissue samples were obtained and analyzed in accordance with the procedures approved by the ethics committee of Stomatological Hospital Shandong University. Four donors were enrolled in the research and the removal of orthodontic teeth from 12-year-old donors was performed with informed consent. The periodontal ligament tissues were then separated, cut into small fragments, and cultured in maintenance medium containing *α*-MEM supplemented with 10% fetal bovine serum (BI). Cells at passage 4 were employed in the present study. Verteporfin was dissolved in PBS.

### 2.2. Lentivirus and Transfection of hPDLSCs

The lentivirus containing the TERT gene was donated by Doctor Li Xiaoyan of the School of Stomatology at Shandong University. hPDLSCs were cultured to 70–80% confluence and then exposed to the viral supernatant for 6 h.

### 2.3. Detection of the Integration of the TERT Gene via the Polymerase Chain Reaction (PCR)

Genomic DNA was isolated using the Wizard® Genomic DNA Purification Kit (Promega, Madison, WI, USA). Construct-specific primers for IRES were used: forward: 5′-TCTCGCCAAAGGAATG-CAAGGTCTGTTG-3′; reverse: 5′-GAGCCATTTGACT CTTTCCACAACTATC-3′.

### 2.4. Detection of mRNA Expression through Real-Time Quantitative (RT-) PCR

Total RNA was extracted from cells using TRIzol according to the manufacturer's protocol (TAKARA, China). Complementary DNA and SYBR Green (TAKARA, China) were employed for RT-PCR. The amplification conditions were as follows: 95°Cfor 15 s, followed by 45 cycles at 60°C for 60 s, and 72°C for 60 s in a LightCycler® 480II (Roche). The primers used for RT-PCR are listed in Table 1.

### 2.5. Detection of Protein Expression via Western Blotting

Total proteins were collected using lysis buffer supplemented with protease inhibitors and phosphatase inhibitors. The protein concentration was determined using the bicinchoninic acid assay kit (Solarbio, Beijing) according to the manufacturer's protocol. Anti-GAPDH was obtained from Protein Tech. The primary antibodies of p21, p16, p53, BAX, MST2, p-LATS, YAP, p-YAP, RUNX2, ALP, ERK, p-ERK, AKT, p-AKT, and COL1 were purchased from CST (Danvers, MA). Anti-BCL-2 was purchased from Wanlei Biotech (Shenyang, China).

### 2.6. Cell Proliferation Assays

The cell counting kit-8 (CCK-8) proliferation assay was performed according to the manufacturer's instructions (Dojindo, Japan). Briefly, 96-well plates were seeded with 1,000 cells per well. Every 24 hours, CCK-8 working solution was added to the wells of the plate. After 2 hours of incubation at 37°C, the plate was subjected to the measurement of spectrophotometric absorbance at a wavelength of 480 nm.

5-Ethynyl-2′-deoxyuridine (EdU) DNA incorporation was performed using an EdU kit (RiboBio, Guangzhou). Cells were treated with EdU working solution at 10 *μ*M in the culture medium for 2 hours in the dark. The cells were then fixed in a solution PBS containing 4% of PFA for 15 minutes. Thereafter, the cells were treated with 2 mg/ml glycine and 0.5% Triton X-100. Next, Hoechst 33342 working solution was added to each well, followed by incubation for 30 minutes in the dark. Images were acquired using an Olympus microscope, and 3 pictures were enrolled in statistical analysis from each group.

### 2.7. Apoptosis Detection Assay

Apoptosis was analyzed based on the cell surface expression of Annexin V-FITC (BestBio, China) according to the manufacturer's instructions. Briefly, approximately 5000 cells were suspended in 400 *μ*l of binding buffer. The cells were then incubated with 5 *μ*l of Annexin V-FITC for 15 minutes in the dark at 4°C. Next, 10 *μ*l of PI staining solution was added, and the cells were placed in the dark at 4°C for 5 minutes. The cells were subsequently analyzed through flow cytometry.

### 2.8. Senescence-Associated *β*-Galactosidase (*β*-GAL) Staining

A Cell Senescence *β*-GAL Staining Kit (Beyotime, China) was employed to detect the cell senescence rate. Cells were washed in PBS and then fixed in a solution containing 4% paraformaldehyde for 20 minutes. Next, the cells were washed in PBS and stained in a *β*-GAL solution. The cells were stained for 24 hours at 37°C without carbon dioxide. Images were acquired using an Olympus microscope. And 3 pictures were enrolled in statistical analysis from each group.

### 2.9. Potential Tumorigenicity Detection Assay

BALB/c mice at the age of seven weeks were provided by the experimental animal center of Shandong University. About 2^∗^10^6^ cells were injected subcutaneously into the BALB/c mice. After 30 days, animals were sacrificed and tumors were removed.

### 2.10. Ethics Statement

This animal experiment has been approved by Stomatological Hospital Shandong University ethics committee. And the research was conducted in accordance with the National Institutes of Health Guidelines for the Care and Use of Laboratory Animals.

### 2.11. Statistical Analysis

The data were analyzed using SPSS 21 software. The results were expressed as the mean ± SD. Student's *t*-test or one-way ANOVA was used to analyze significant differences between groups. *P* < 0.05 indicates a significant difference.

## 3. Results

### 3.1. Immortalization of hPDLSCs

**Figure 1:**
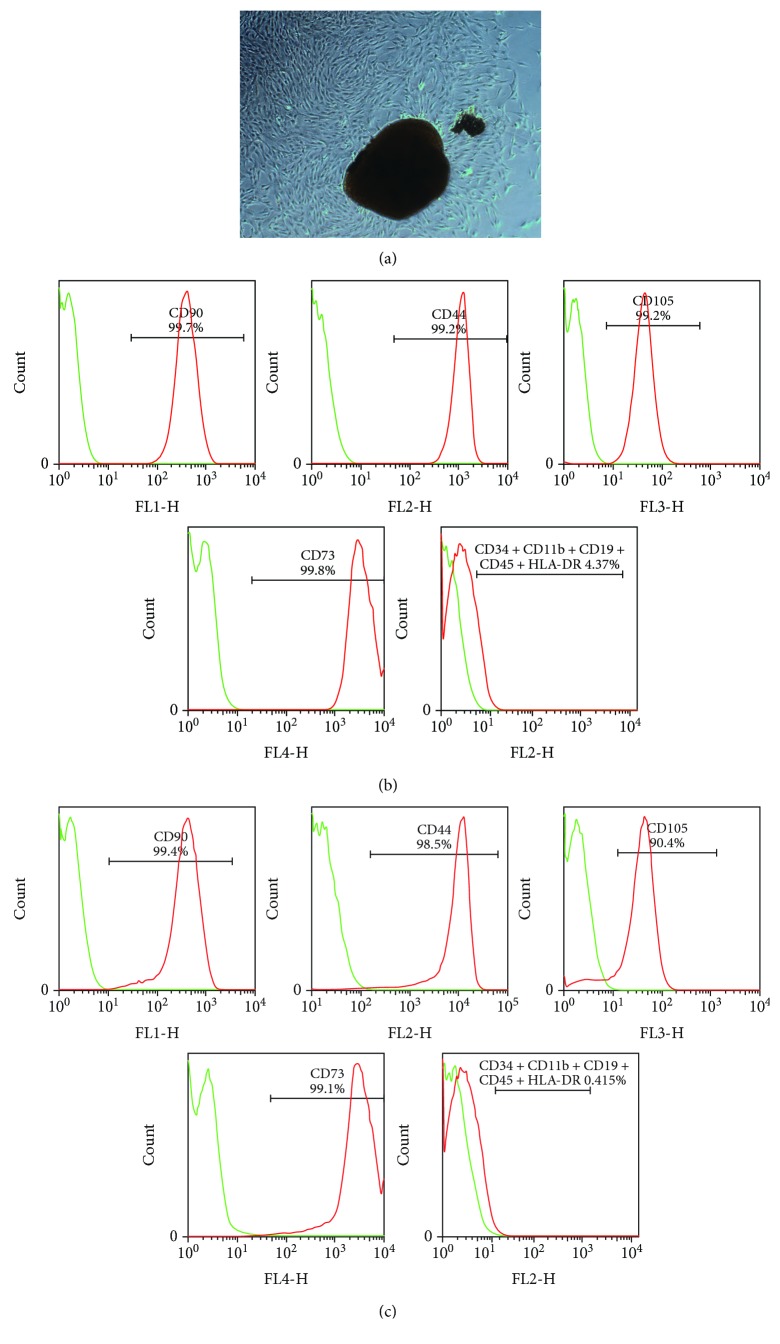
Assays of hPDLSCs and hPDLSC-TERT characteristics. (a) hPDLSCs under a phase contrast microscope. (b, c) Flow cytometry analysis showed that hPDLSCs expressed CD73, CD44, CD90, and CD105 but did not express CD34, CD11b, CD19, CD45, and HLA-DR. Scale bar = 200 *μ*m.

**Figure 2:**
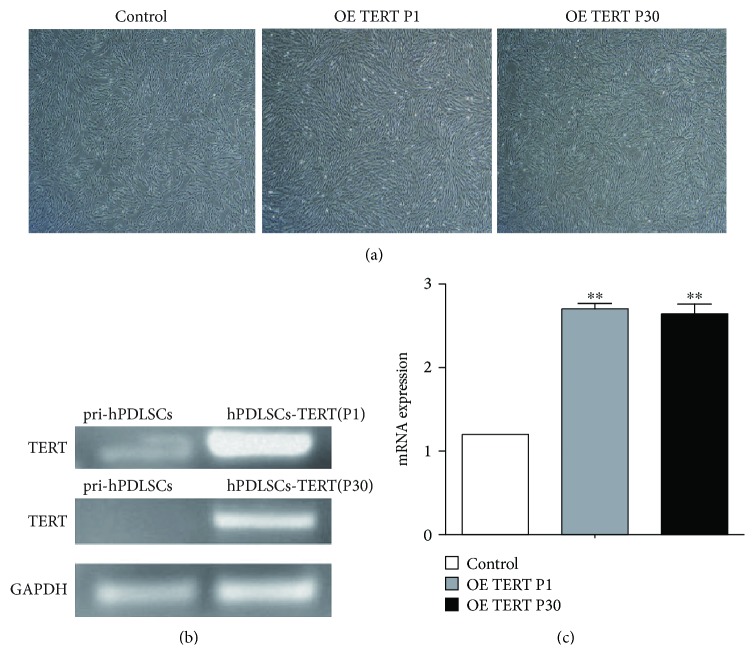
Cell morphology and detection of TERT expression levels. (a) Morphology of hPDLSCs at passage four and TERT-hPDLSCs at passages one and thirty. (b) Identification of TERT gene integration in TERT-hPDLSCs with genomic DNA. (c) The expression level of TERT was detected via RT-PCR. TERT was increased over 100% after gene overexpression in hPDLSCs. Scale bar = 200 *μ*m. ^∗^*P* < 0.05, ^∗∗^*P* < 0.01.

First, hPDLSCs were isolated from four 12-year-old donors' molars, followed by culture in *α*-MEM medium, and flow cytometry was employed to identify the characteristics of hPDLSCs and hPDLSCs transfected with lentivirus containing the hTERT gene (TERT-hPDLSCs) (Figure 1). Following transfection with the lentivirus containing hTERT, the cells bypassed senescence and grew for over 50 population doublings. In contrast, the original primary cells entered crisis after approximately 15 population doublings. Higher expression of hTERT was confirmed in TERT-hPDLSCs in comparison with primary cells via both PCR and RT-PCR analyses (Figure 2). The expression level of TERT gene was increased about 100% after gene overexpression in PDLSCs.

### 3.2. Increased Proliferation Ability of TERT-hPDLSCs

**Figure 3:**
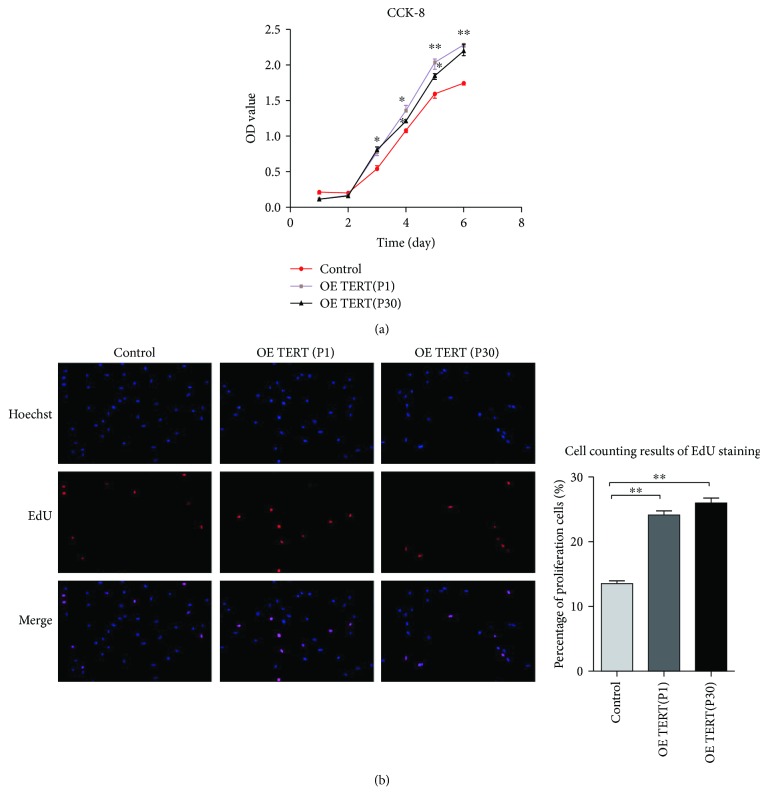
The proliferation ability of TERT-hPDLSCs is increased. (a) Cell proliferation activity was detected by CCK-8 assay; hTERT-PDLSCs (P1) (2.28 ± 0.01) and hTERT-PDLSCs (P30) (2.21 ± 0.03) showed higher proliferation activity at day 6 than the control group (1.74 ± 0.01). (b) EdU staining showed that the percentage of proliferation cell in hTERT-PDLSCs (P1) and hTERT-PDLSCs (P30) was higher than the control group. Scale bar = 200 *μ*m.^∗^*P* < 0.05, ^∗∗^*P* < 0.01.

The proliferation of both hPDLSCs and TERT-hPDLSCs appeared stable in early passages, but TERT-hPDLSCs displayed a significantly more rapid growth rate than primary cells. CCK-8 assay showed that TERT-hPDLSCs (P1, P30) exhibited a higher proliferation activity than the control group (2.28 ± 0.01, 2.21 ± 0.03 versus 1.74 ± 0.01) (Figure 3(a)). The results of EdU staining showed the same effect (Figures 3(b)).

### 3.3. Lower Senescence and Apoptosis Rates of TERT-hPDLSCs

**Figure 4:**
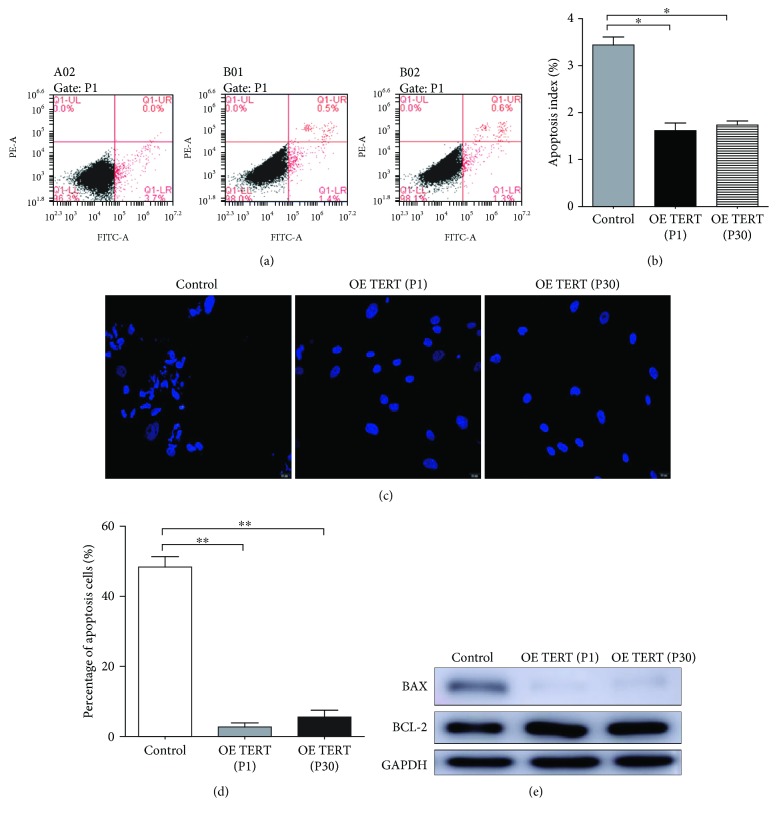
The apoptosis rate of TERT-hPDLSCs is decreased. (a, b) Flow cytometry and (c, d) TUNEL staining were employed to assay the apoptosis rate in hPDLSCs and TERT-hPDLSCs, which revealed a lower apoptosis rate of TERT-hPDLSCs than that of hPDLSCs. (e) Western blot showed that overexpression of TERT gene in PDLSCs led to proapoptosis protein BAX decrease, while antiapoptotic protein BCL-2 increases. Scale bar = 100 *μ*m.^∗^*P* < 0.05, ^∗∗^*P* < 0.01.

**Figure 5:**
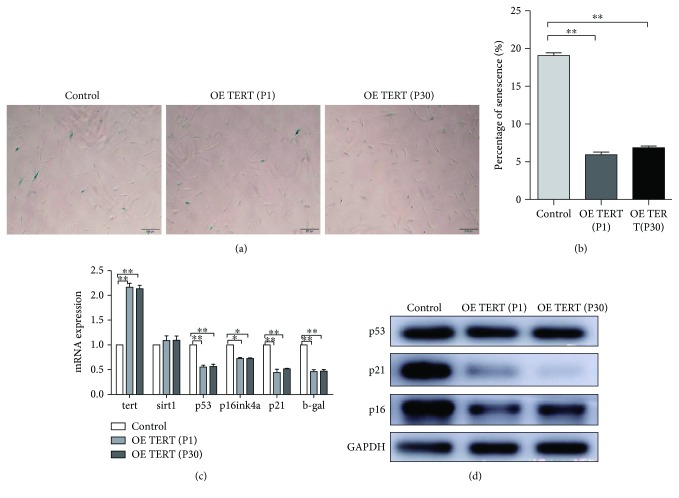
The senescence rate of TERT-hPDLSCs is decreased. (a, b) Cell senescence was detected using the *β*-GAL staining assay, which revealed a significantly decreased cell senescence rate of TERT-hPDLSCs compared with that of hPDLSCs. (c) The expression levels of genes associated with cell senescence were assessed using RT-PCR and were found to be decreased after TERT was overexpressed. (d) Proteins related to senescence decreased in TERT-PDLSCs. Scale bar = 200 *μ*m.^∗^*P* < 0.05, ^∗∗^*P* < 0.01.

The apoptosis rate was tested through flow cytometry (Figures 4(a) and 4(b)) and TUNEL staining (Figures 4(c) and 4(d)). The results showed that TERT-hPDLSCs exhibited fewer apoptotic cells than hPDLSCs. Western blotting results revealed that overexpression of the TERT gene led to the increase of BCL-2 protein and decrease of BAX protein, which could explain the changing of apoptotic cells. We also examined senescence via *β*-GAL staining in hPDLSCs and TERT-hPDLSCs at the early and late passages. As shown in the counting results, the rate of TERT-hPDLSC stained cells was lower than that of the control group (18.75+3.73)% in both the early (6.90+0.84)% and late passages (7.49+0.74)% (Figures 5(a) and 5(b)). Next, we detected senescence-related genes through RT-PCR. All the genes showed a lower expression level in TERT-hPDLSCs than that in hPDLSCs (Figure 5(c)). Proteins related to cell senescence also showed lower expression level than the control group, like p53, p16, and p21, detected by Western blotting (Figure 5(d)).

### 3.4. Maintenance of Osteogenesis Ability in TERT-hPDLSCs

**Figure 6:**
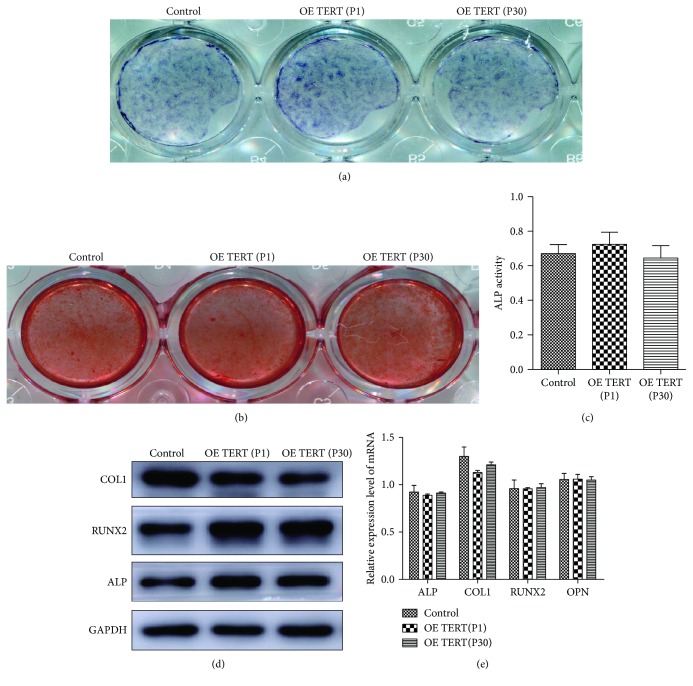
The osteogenic differentiation ability of TERT-hPDLSCs is maintained. (a, b) Calcified sediment around cells was stained with Alizarin red (21 days) and ALP (15 days) after osteogenesis induction. TERT-hPDLSCs showed similar osteogenic differentiation ability to hPDLSCs even at passage thirty. (c) 15 days after being osteogenically induced, ALP activity was quantitatively analyzed by ALP activity assay kit. (d, e) The expression levels of osteogenic differentiation markers were examined using RT-PCR and Western blotting. The results showed no difference between the TERT overexpression group and the control group.

The osteogenesis function was tested via ALP and Alizarin red staining after the cells in every group were cultured in osteogenic induction medium for 28 days (Figures 6(a) and 6(b)). The osteogenesis ability of TERT-hPDLSCs showed no obvious decrease compared with that of the control group. ALP activity assay showed that the osteogenesis ability of TERT-PDLSCs (P1) (0.72 ± 0.05) and TERT-PDLSCs (P30) (0.66 ± 0.05) was similar to the control group (0.69 ± 0.050) after osteogenesis induction for 15 days (Figure 6(c)). Next, we detected the expression of osteogenesis-related proteins and genes which were extracted from samples after 21 days of osteogenesis induction; the results showed no significant difference (Figures 6(d) and 6(e)).

### 3.5. Potential Tumorigenicity of TERT-hPDLSCs in Nude Mice

**Figure 7:**
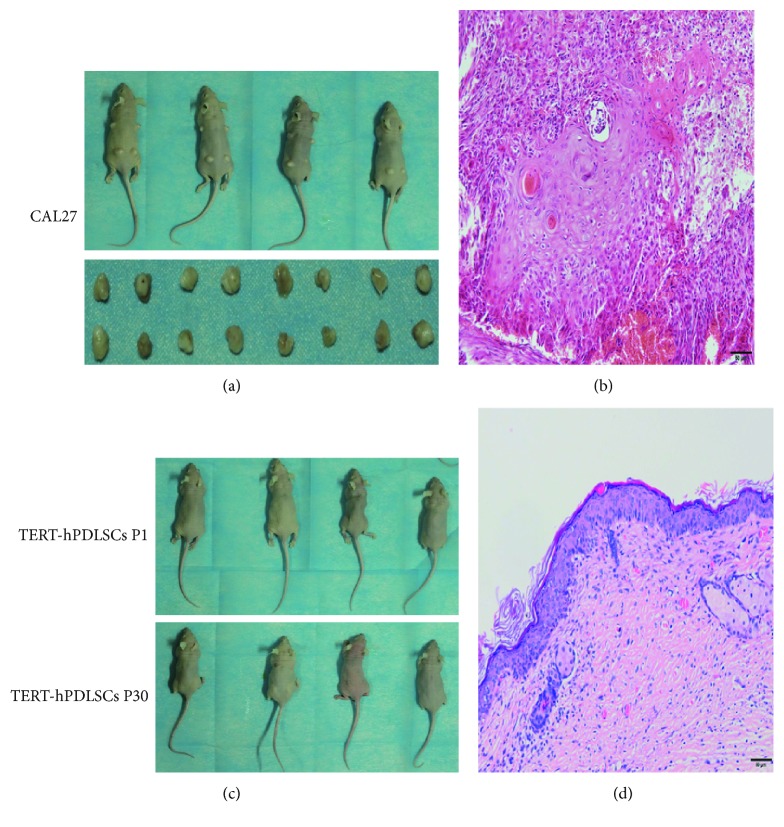
TERT-hPDLSCs show no tumorigenicity in nude mice. The tumorigenicity experiments of TERT-hPDLSCs. Cells were subcutaneously injected into CB17 SCID nude mice and examined after 5 weeks. (a) Cal27 cells served as a positive control. (b) The HE staining showed that there were undifferentiated cells in the neoplasm. (c) TERT-hPDLSCs at passages one and thirty injected show no tumorigenicity. (d) HE staining showed that it was normal epithelial tissues.

BALB/c mice at the age of five weeks were used to test the potential tumorigenicity of TERT-hPDLSCs in the first and thirtieth passages. The mice were divided into three groups, with six mice per group. The TERT-hPDLSCs (P1), TERT-hPDLSCs (P30), and CAL27 cells (oral squamous cells) were subcutaneously injected on both dorsal sides of nude mice. Tumor formation was observed for 5 weeks after injection. Tumors were found in the mice after CAL27 cells (oral squamous cells) were embedded (Figure 7(a)), and HE staining showed that there were undifferentiated cells in the tumor (Figure 7(b)). At the same time, no tumor was found in the TERT-hPDLSC groups (Figures 7(c) and 7(d)), which means the cell of TERT-hPDLSCs did not have the potential tumorigenicity.

### 3.6. Expression Levels of Proteins in the Hippo/YAP Pathway

**Figure 8:**
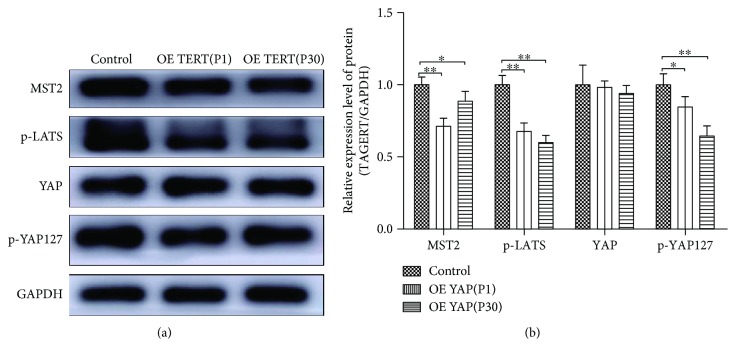
The Hippo/YAP signaling pathway was inactivated in TERT-hPDLSCs. After TERT was overexpressed, the expression levels of MST2, p-LATS, and p-YAP127 were decreased, as detected via Western blotting. The expression level of YAP in TERT-hPDLSCs was similar to the control group, which meant overexpression of TERT led to the dephosphorylation of YAP. ^∗^*P* < 0.05, ^∗∗^*P* < 0.01.

Western blotting was employed to determine whether the expression levels of key proteins were altered after TERT overexpression (Figures 8(a) and 8(b)). The results showed that the upstream kinases like MST2 and p-LATS were decreased after TERT overexpression, which led to the decrease of p-YAP. At the same time, the expression of total YAP proteins in TERT-hPDLSCs displays similar level with the control group cell, which means activated form of YAP were increased in TERT-hPDLSCs.

### 3.7. YAP Inhibition Led to Decrease of Cell Proliferation Ability

**Figure 9:**
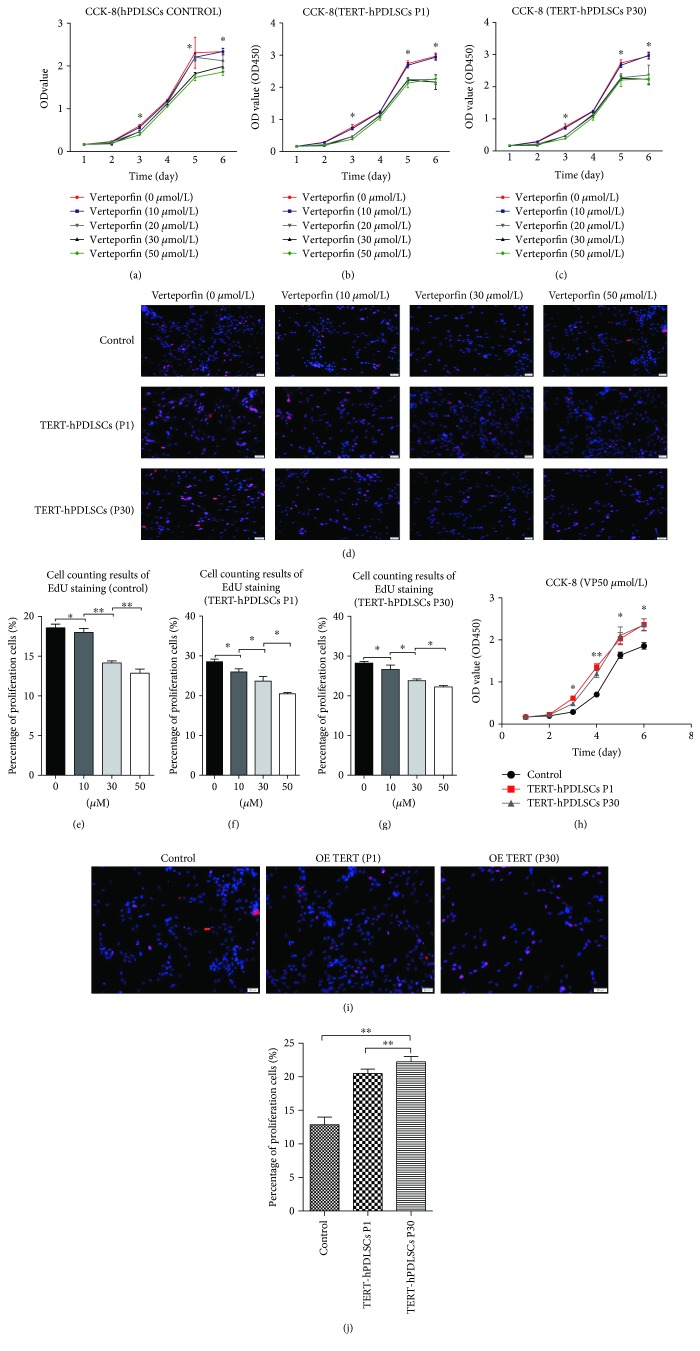
Verteporfin led to a decreased proliferation capacity. Cell proliferation ability was detected after verteporfin was used to inhibit the binding of YAP to downstream transfactor TEAD. (a-c) CCK-8 tests showed that with the increase of verteporfin, the proliferation activity of the TERT-hPDLSCs (P1, P30) and control group cells was both decreased. (d-g) EdU staining assay confirmed the results of CCK-8. (h-j) Overexpression of TERT could abrogate the inhibition of proliferation activity caused by verteporfin. Scale bar = 200 *μ*m. ^∗^*P* < 0.05, ^∗∗^*P* < 0.01.

We used verteporfin to inhibit the binding of YAP to downstream TEAD. Treatment was performed using different concentrations of verteporfin for 72 h. CCK-8 (Figures 9(a)–9(c)) and EdU assays (Figures 9(d)–9(g)) showed that with the increase of verteporfin, the proliferation activity of the TERT-hPDLSCs (P1, P30) and control group cells was both decreased. Next, the proliferation ability of TERT-hPDLSCs was assessed through EdU and CCK-8 assays after YAP inhibition as well as by cell counting in the 50 *μ*mol/l treatment group. The results showed that TERT-hPDLSCs grew more slowly but still better than hPDLSCs in the presence of the same concentration of verteporfin (Figures 9(h)–9(j)). These results indicated that the increasing proliferation activity led by TERT overexpression was mediated by Hippo-YAP signal.

### 3.8. YAP Inhibition Increased the Cell Senescence Rate

**Figure 10:**
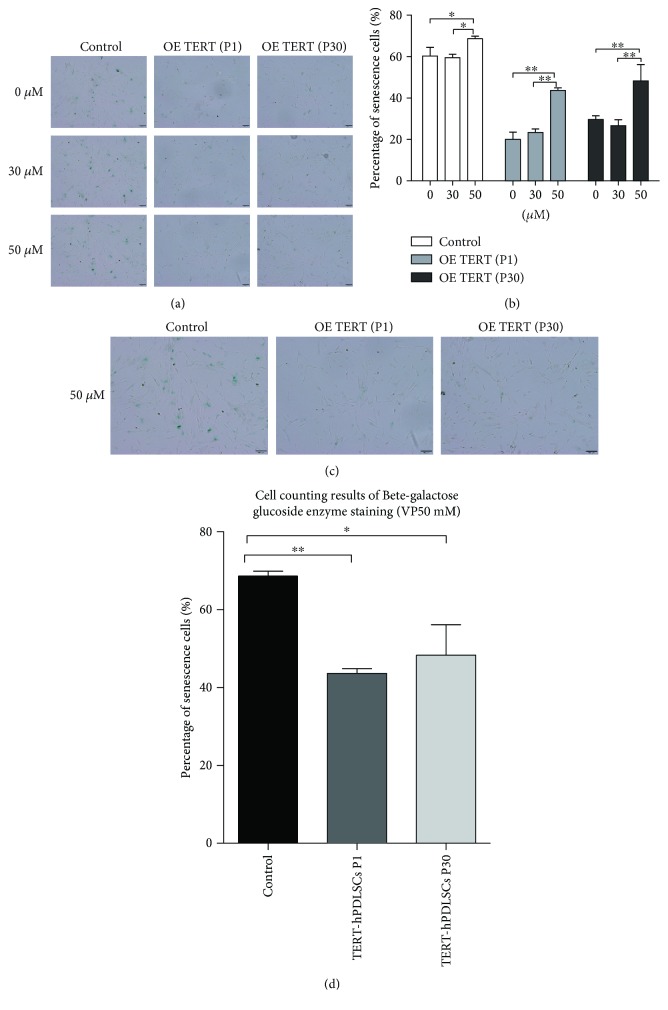
Verteporfin led to increase senescence cell rate. The rate of cell senescence was assayed via *β*-GAL staining after verteporfin treatment. The number of stained cells was counted when treatment with verteporfin was performed at different concentration. (a, b) The cell counting results showed that the cell senescence rate gradually amplified with the increase of verteporfin. (c, d) At the concentration of 50 *μ*M of verteporfin, the TERT-hPDLSC senescence rate was lower than that of hPDLSCs. Scale bar = 200 *μ*m. ^∗^*P* < 0.05, ^∗∗^*P* < 0.01.

The *β*-GAL staining assay was employed to assess the senescence rate after YAP was inhibited with verteporfin (0, 10, 30, and 50 *μ*mol/l) for 72 h (Figures 10(a) and 10(b)). The senescence rate was increased with the increase of verteporfin, which means the Hippo-YAP signal was involved in cell senescence (Figures 10(a) and 10(b)). From the result of Figure 10(b), we can also conclude that overexpression of TERT could abolish cell senescence caused by verteporfin. At the concentration of 50 *μ*M, the senescence rate of TERT-hPDLSCs was still lower than that of the control group (Figures 10(c) and 10(d)). These results indicated that overexpression of the TERT gene in hPDLSCs could lessen cell senescence, and YAP was involved in the progress.

### 3.9. ERK and AKT Signal Pathway May Involve in the Hippo-YAP Signal Pathway

**Figure 11:**
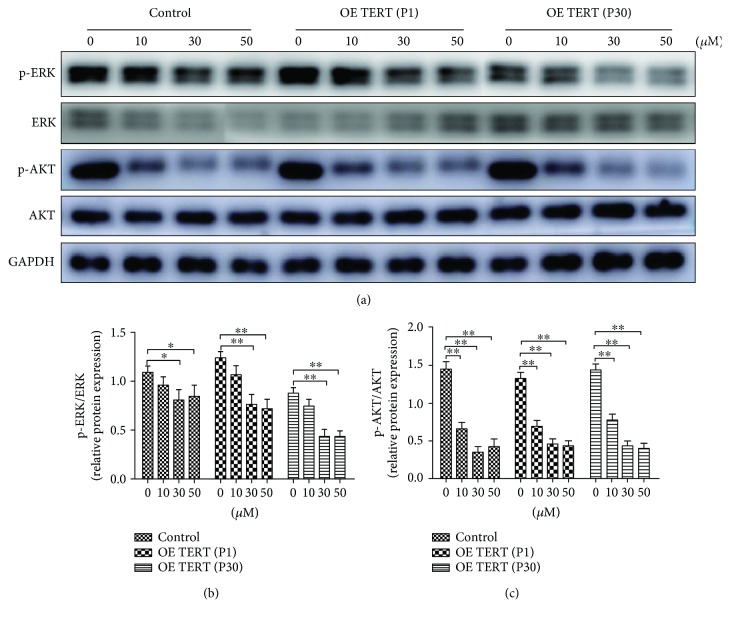
Verteporfin decreases AKT and ERK expression levels. Western blotting was employed to detect the expression levels of AKT and ERK. (a, b) p-ERK and (a, c) p-AKT expression levels were found to be decreased after verteporfin treatment. ^∗^*P* < 0.05, ^∗∗^*P* < 0.01.

ERK [21, 22] and AKT [23, 24] signal pathways were related to multiple kinds of physiological functions. Our results showed that p-AKT and p-ERK expression levels were found to be decreased after verteporfin increases, in a dose-dependent manner, while the total ERK and AKT show no obvious changes (Figure 11). These results indicated that YAP could regulate the phosphorylation state of ERK and AKT signal.

## 4. Discussion

Once regarded as a significant gene, the role of TERT is to relengthen telomeres. TERT is now also considered a multifunctional protein [25]. It is accepted that TERT is upregulated in pluripotent stem cells and supports cell self-renewal by maintaining telomere length. TERT can prevent the end structure of telomeres from being gradually lost with cell divisions to maintain telomere length [26]. However, other regulatory roles of TERT have been less well described, such as how it controls cell senescence and whether it may engage in crosstalk with other pathways.

The aim of this study was to extend the length of telomeres, which gradually shorten with cell division to delay cell senescence. At the same time, stem cell characteristics were maintained. Then, we explored whether the Hippo/YAP signaling pathway mediated the establishment of the immortalized hPDLSC line and whether it was involved in the regulation of the biological behaviors of proliferation and senescence in the cell line.

We showed that a high level of telomerase activity was maintained in hPDLSCs at both early and late passages after hPDLSCs were transfected with lentivirus containing the TERT gene. Thus, we successfully created an immortalized hPDLSC line that maintained most of the characteristics of the primary hPDLSCs. The TERT-hPDLSCs showed a similar osteogenesis differentiation ability to primary hPDLSCs *in vitro* in both the first and thirtieth passages. Furthermore, the TERT-hPDLSCs displayed a stronger proliferation ability, similar osteogenic differentiation ability, and lower apoptosis or senescence rates compared with primary hPDLSCs. The hPDLSC-TERT cell line also showed no tumorigenicity in nude mice. These data indicated that the hPDLSC-TERT cell overcomes the problem of reproductive senescence of hPDLSCs when cultured *in vitro*. Additionally, it presents similar or improved biological characteristics compared with the primary hPDLSCs and could serve as a useful tool to mechanistic and functional studies. Our results showed decrease expression of some key protein in the Hippo/YAP signaling pathway, such as MST2, p-LATS, and p-YAP in TERT-hPDLSCs. This finding indicated that TERT leads to inactivation of the Hippo signaling pathway and the dephosphorylation of YAP. In other words, overexpression of TERT may activate YAP and enhance the function of the pathway, particularly regarding the enhancement of cell proliferation and inhibition of apoptosis and cell senescence.

Verteporfin is a type of drug employed as photodynamic therapy for the treatment of macular degeneration that eliminates abnormal blood vessels. Verteporfin has also been found to abolish liver hyperplasia induced by YAP overexpression [27]. These studies indicated that verteporfin inhibited the combination of YAP and TEAD.

A certain concentration of verteporfin can prevent YAP from binding to TEAD in the nucleus; thus, YAP cannot activate its downstream genes to accomplish the regulation of biological behaviors in various cell types [28]. We used verteporfin to inhibit the binding of YAP to downstream TEAD. Additionally, we detected proliferation using the CCK-8 and EdU assays. The CCK-8 results showed that the proliferation capacity gradually slowed down from the third day after verteporfin treatment. The primary hPDLSCs showed the same downward trend but exhibited a lower proliferation rate than TERT-hPDLSCs. The counting results obtained following EdU staining showed that after verteporfin treatment, the cell proliferation rate decreased. The cell proliferation rate of primary hPDLSCs in the control group also decreased after verteporfin treatment, but the rate was lower than that of TERT-hPDLSCs. We detected cell senescence using the *β*-GAL staining assay, and the results showed an increased senescence cell rate after verteporfin treatment. The primary hPDLSCs exhibited a higher rate of cell senescence than TERT-hPDLSCs.

ERK and AKT are intracellular signal pathways which are involved in many fundamental cellular processes, such as cell differentiation and proliferation. In our study, we found that with the increase of verteporfin, p-ERK and p-AKT decreased gradually, in a dose-dependent manner. But the total ERK and AKT remained unchanged, which means YAP-TEAD complex could affect the phosphorylation of ERK and AKT. But the exact mechanism remains to be further studied.

Therefore, verteporfin inhibited the function of YAP, which can inhibit cell proliferation and lead to senescence. Under the same inhibition conditions, the proliferation ability of TERT-hPDLSCs remained higher and the senescence rate remained lower than those of hPDLSCs. These data confirmed that TERT may affect the Hippo/YAP signaling pathway and control hPDLSCs proliferation and senescence through the Hippo/YAP signaling pathway.

## 5. Conclusion

In this study, we showed that we could establish an immortalized PDLSC line through transfection of hPDLSCs with lentivirus containing the TERT gene. We confirmed that TERT can control hPDLSC senescence through the Hippo/YAP signal pathway. The Hippo/YAP signal pathway could mediate the establishment of the immortalized PDLSC line. This research may provide a widely available and accessible source of hPDLSCs to study periodontal tissue regeneration and offer a theoretical basis for research on applications of this cell line in the future.

## Figures and Tables

**Table 1:** Primers for quantitative PCR.

GENE	Forward primer	Reverse primer
*TERT*	5′-CCGATTGTGAACATGGACTACG-3′	5′-CACGCTGAACAGTGCCTTC-3′
*ALP*	*5*′*-*CCACGTCTTCACATTTGGTG-3′	5′- AGACTGCGCCTGGTAGTTGT -3′
*COL1*	*5*′*-GCTGATGATGCCAATGTGGTT-3*′	*5*′*-CCAGTCAGAGTGGCACATCTTG-3*′
*RUNX2*	*5*′*-GTTTCACCTTGACCATAACCGT-3*′	*5*′*-GGGACACCTACTCTCATACTGG-3*′
*OPN*	*5*′*- AATCCGGACTGTGACGAGTTG-3*′	*5*′*- CAGCAGAGCGACACCCTAGAC-3*′
*SIRT1*	5′-ATTTTCCATGGCGCTGAGGTA-3′	5′-TCAGTCCCAAATCCAGCTCC-3′
*P53*	5′-CAGCACATGACGGAGGTTGT-3′	5′-TCATCCAAATACTCCACACGC-3′
*P16INK4A*	5′-ACCGCTTCTGCCTTTTCACT-3′	5′-CCCCTGAGCTTCCCTAGTTC-3′
*P21*	5′-CGATGGAACTTCGACTTTGTCA-3′	5′-GCACAAGGGTACAAGACAGTG-3′
*Β-GAL*	5′-TATACTGGCTGGCTAGATCACTG-3′	5′-GGCAAAATTGGTCCCACCTATAA-3′
*YAP*	5′-AATGACGACCAATAGCTCAGATCC-3′	5′-CACTGTAGCTGCTCATGCTYAGTCC-3
*GAPDH*	5′-AGAAGGCTGGGGCTCA7ITITIG-3′	5′-AGGGGCCATCCACAGTCTTC-3′

## Data Availability

The data used to support the findings of this study are available from the corresponding author upon request.

## References

[1] Ding G., Liu Y., Wang W. (2010). Allogeneic periodontal ligament stem cell therapy for periodontitis in swine. *Stem Cells*.

[2] Kalra N., Pradeep A. R., Priyanka N., Kumari M. (2013). Association of stem cell factor and high-sensitivity C reactive protein concentrations in crevicular fluid and serum in patients with chronic periodontitis with and without type 2 diabetes. *Journal of Oral Science*.

[3] Zhu S. Y., Wang P. L., Liao C. S. (2017). Transgenic expression of ephrinB2 in periodontal ligament stem cells (PDLSCs) modulates osteogenic differentiation via signaling crosstalk between ephrinB2 and EphB4 in PDLSCs and between PDLSCs and pre-osteoblasts within co-culture. *Journal of Periodontal Research*.

[4] Abd Rahman F., Mohd Ali J., Abdullah M., Abu Kasim N. H., Musa S. (2016). Aspirin enhances steogenic potential of periodontal ligament stem cells (PDLSCs) and modulates the expression profile of growth factor-associated genes in PDLSCs. *Journal of Periodontology*.

[5] Vono R., Jover Garcia E., Spinetti G., Madeddu P. (2018). Oxidative stress in mesenchymal stem cell senescence: regulation by coding and noncoding RNAs. *Antioxidants & Redox Signaling*.

[6] Son M. J., Kwon Y., Son T., Cho Y. S. (2016). Restoration of mitochondrial NAD(+) levels delays stem cell senescence and facilitates reprogramming of aged somatic cells. *Stem Cells*.

[7] Stockklausner C., Raffel S., Klermund J. (2015). A novel autosomal recessive TERT T1129P mutation in a dyskeratosis congenita family leads to cellular senescence and loss of CD34+ hematopoietic stem cells not reversible by mTOR-inhibition. *Aging*.

[8] Kim H., Ahn D., Sohn J. H., Kim Y. H., Lee J. H., Lee H. (2018). TERT promoter mutation and telomere length in salivary gland tumors. *Pathology & Oncology Research*.

[9] Lee Y., Koh J., Kim S. I. (2017). The frequency and prognostic effect of TERT promoter mutation in diffuse gliomas. *Acta Neuropathologica Communications*.

[10] Pramanik M. M. D., Nagode S. B., Kant R., Rastogi N. (2017). Visible light catalyzed Mannich reaction between tert-amines and silyl diazoenolates. *Organic & Biomolecular Chemistry*.

[11] Koehne I., Bachmann S., Niklas T., Herbst-Irmer R., Stalke D. (2017). A novel bulky heteroaromatic-substituted methanide mimicking NacNac: Bis(4,6-tert-butylbenzoxazol-2-yl)methanide in s-block metal coordination. *Chemistry*.

[12] Eskandani M., Hamishehkar H., Ezzati Nazhad Dolatabadi J. (2014). Cytotoxicity and DNA damage properties of tert-butylhydroquinone (TBHQ) food additive. *Food Chemistry*.

[13] Ahmad F., Patrick S., Sheikh T. (2017). Telomerase reverse transcriptase (TERT) - enhancer of zeste homolog 2 (EZH2) network regulates lipid metabolism and DNA damage responses in glioblastoma. *Journal of Neurochemistry*.

[14] Bai X., Kong Y., Chi Z. (2017). *MAPK* pathway and *TERT* promoter gene mutation pattern and its prognostic value in melanoma patients: a retrospective study of 2,793 cases. *Clinical Cancer Research*.

[15] Xu W., Li F., Xu Z., Sun B., Cao J., Liu Y. (2017). Tert-butylhydroquinone protects PC12 cells against ferrous sulfate-induced oxidative and inflammatory injury via the Nrf2/ARE pathway. *Chemico-Biological Interactions*.

[16] Yu F. X., Zhao B., Panupinthu N. (2012). Regulation of the Hippo-YAP pathway by G-protein-coupled receptor signaling. *Cell*.

[17] Aragona M., Panciera T., Manfrin A. (2013). A mechanical checkpoint controls multicellular growth through YAP/TAZ regulation by actin-processing factors. *Cell*.

[18] Park H. W., Kim Y. C., Yu B. (2015). Alternative Wnt signaling activates YAP/TAZ. *Cell*.

[19] Azzolin L., Panciera T., Soligo S. (2014). YAP/TAZ incorporation in the *β*-catenin destruction complex orchestrates the Wnt response. *Cell*.

[20] Yin Z., Wang Q., Li Y., Wei H., Shi J., Li A. (2016). A novel method for banking stem cells from human exfoliated deciduous teeth: lentiviral TERT immortalization and phenotypical analysis. *Stem Cell Research & Therapy*.

[21] Cargnello M., Roux P. P. (2011). Activation and function of the MAPKs and their substrates, the MAPK-activated protein kinases. *Microbiology and Molecular Biology Reviews*.

[22] Sun Y., Liu W. Z., Liu T., Feng X., Yang N., Zhou H. F. (2015). Signaling pathway of MAPK/ERK in cell proliferation, differentiation, migration, senescence and apoptosis. *Journal of Receptor and Signal Transduction Research*.

[23] Schiaffino S., Mammucari C. (2011). Regulation of skeletal muscle growth by the IGF1-Akt/PKB pathway: insights from genetic models. *Skeletal Muscle*.

[24] Sittewelle M., Monsoro-Burq A. H. (2018). AKT signaling displays multifaceted functions in neural crest development. *Developmental Biology*.

[25] Wilson R., Urraca N., Skobowiat C. (2015). Assessment of the tumorigenic potential of spontaneously immortalized and hTERT-immortalized cultured dental pulp stem cells. *Stem Cells Translational Medicine*.

[26] Ikbale E. A., Goorha S., Reiter L. T., Miranda-Carboni G. A. (2016). Effects of hTERT immortalization on osteogenic and adipogenic differentiation of dental pulp stem cells. *Data in Brief*.

[27] Feng J., Gou J., Jia J., Yi T., Cui T., Li Z. (2016). Verteporfin, a suppressor of YAP-TEAD complex, presents promising antitumor properties on ovarian cancer. *OncoTargets and Therapy*.

[28] Konstantinou E. K., Notomi S., Kosmidou C. (2017). Verteporfin-induced formation of protein cross-linked oligomers and high molecular weight complexes is mediated by light and leads to cell toxicity. *Scientific Reports*.

